# Mutation Patterns of 16 Genes in Primary and Secondary Acute Myeloid Leukemia (AML) with Normal Cytogenetics

**DOI:** 10.1371/journal.pone.0042334

**Published:** 2012-08-09

**Authors:** Marta Fernandez-Mercado, Bon Ham Yip, Andrea Pellagatti, Carwyn Davies, María José Larrayoz, Toshinori Kondo, Cristina Pérez, Sally Killick, Emma-Jane McDonald, María Dolores Odero, Xabier Agirre, Felipe Prósper, María José Calasanz, James S. Wainscoat, Jacqueline Boultwood

**Affiliations:** 1 LLR Molecular Haematology Unit, NDCLS, John Radcliffe Hospital, Oxford, United Kingdom; 2 Department of Genetics, University of Navarra, Pamplona, Spain; 3 Division of Hematology, Kawasaki Medical School, Okayama, Japan; 4 Laboratory of Myeloproliferative Syndromes, Oncology Area, Foundation for Applied Medical Research, Clínica Universitaria, Universidad de Navarra, Pamplona, Spain; 5 Department of Haematology, Royal Bournemouth Hospital, Bournemouth, United Kingdom; 6 Division of Oncology, Center for Applied Medical Research, Universidad de Navarra, Pamplona, Spain; 7 Division of Cancer and Area of Cell Therapy and Hematology Service, Foundation for Applied Medical Research, Clínica Universitaria, Universidad de Navarra, Pamplona, Spain; University of Texas MD Anderson Cancer Center, United States of America

## Abstract

Acute myeloid leukemia patients with normal cytogenetics (CN-AML) account for almost half of AML cases. We aimed to study the frequency and relationship of a wide range of genes previously reported as mutated in AML (*ASXL1*, *NPM1*, *FLT3*, *TET2*, *IDH1*/*2*, *RUNX1*, *DNMT3A*, *NRAS*, *JAK2*, *WT1*, *CBL*, *SF3B1*, *TP53*, *KRAS* and *MPL*) in a series of 84 CN-AML cases. The most frequently mutated genes in primary cases were *NPM1* (60.8%) and *FLT3* (50.0%), and in secondary cases *ASXL1* (48.5%) and *TET2* (30.3%). We showed that 85% of CN-AML patients have mutations in at least one of *ASXL1*, *NPM1*, *FLT3*, *TET2*, *IDH1/2* and/or *RUNX1*. Serial samples from 19 MDS/CMML cases that progressed to AML were analyzed for *ASXL1/TET2/IDH1/2* mutations; seventeen cases presented mutations of at least one of these genes. However, there was no consistent pattern in mutation acquisition during disease progression. This report concerns the analysis of the largest number of gene mutations in CN-AML studied to date, and provides insight into the mutational profile of CN-AML.

## Introduction

Acute myeloid leukemia (AML) is a heterogeneous disease in terms of karyotype and molecular abnormalities. The discovery of the classic karyotype abnormalities in AML such as the t(15;17) has been invaluable in enabling more accurate prognostic estimates, the development of specific therapies and the molecular monitoring of disease. However, approximately half of AML patients have no karyotype abnormality (CN-AML). This group of AML cases is presumably heterogeneous in all respects, and molecular monitoring is not possible unless there is an associated mutation. Recently it has been demonstrated that mutations of *FLT3*, *NPM1* and *CEBPA* genes are preferentially found in CN-AML. [1] Nevertheless many cases do not possess such mutations and this imposes a severe limitation in understanding their specific pathophysiology and monitoring disease progression. We have chosen to study CN-AML with the aim of finding a restricted panel of genes which are mutated in the majority of cases. In a series of 84 CN-AML patients, we examined 16 genes with mutations that had previously been described in cases of CN-AML (Table S1). [2], [3], [4], [5], [6], [7], [8], [9], [10], [11], [12], [13], [14], [15], [16], [17] The characterisation of cases by the presence or absence of mutations in these selected genes should allow a molecular dissection of cases of CN-AML into different biological and prognostic groups, as well as achieving the long sought after goal of molecular monitoring of CN-AML.

## Design and Methods

### Patients

A total of 84 AML patients (mean age 64, range 16 to 86, 23 patients under 60; 52 male, 32 female) with no cytogenetic abnormalities were recruited for mutational analysis, including 51 primary cases (mean age 60, range 16 to 86, 20 patients under 60; 27 male, 24 female) and 33 cases secondary to either MDS (n = 24) or CMML (n = 9) (mean age 70, range 51 to 81, 3 patients under 60; 25 male, 8 female). The karyotype was investigated again at the time of transformation in 31 of the 33 secondary cases, and found to be normal. An additional 100 cases were investigated for mutations in *ASXL1* from AML patients showing different karyotypic abnormalities. Some of the cases included in the present study (16 CN-AML and 51 cases with aberrant cytogenetics) have been previously analyzed for ASXL1 exon 12 mutations, and results reported elsewhere. [18] All karyotypes were analyzed by conventional G-banding in at least 30 metaphases. Samples showing inv(16), t(8;21) or t(15;17) at karyotype were subjected to confirmation by molecular techniques. This study was approved by the ethics committees of the institutes involved: the John Radcliffe Hospital (Oxford 06/Q1606/110), the Royal Bournemouth Hospital (Bournemouth 9991/03/E) and the University of Navarre (Pamplona IRB00006933); written informed consent was received from all patients.

### DNA sequencing and analysis

Genomic DNA was isolated from patient bone marrow or peripheral blood samples. Primers and PCR conditions for the 16 genes analyzed are detailed in Table S2. Relevant regions were selected for analysis (Table S2): exons 12 of *ASXL1* (NM_015338.5) and *NPM1* (NM_002520), exons 11 and 17 of *FLT3* (NM_004119), exon 14 of *JAK2* (NM_004972), entire coding region of *TET2* (NM_001127208.2), Exons 4 of *IDH1* (NM_005896) and *IDH2* (NM_002168), exons 3 to 8 of *RUNX1* (NM_001001890), exons 7–9 of *CBL* (NM_005188), exons 9 and 10 of *MPL* (NM_005373), exons 3 to 9 of *TP53* (NM_000546), exons 2 and 3 of *NRAS* (NM_002524.4) and *KRAS* (NM_033360), Exons 4 to 9 of *WT1* (NM_024426), exons 7 to 23 of *DNMT3A* (NM_022552) and exons 12 to 16 of *SF3B1* (NM_012433.2). PCR was performed using ThermoStart PCR Master Mix (Thermo Fisher Scientific), following the manufacturer's protocol. PCR products were purified and bidirectionally sequenced using the BigDye Terminator v1.1 cycle sequencing kit (Applied Biosystems, Foster City, CA, USA) and an ABI 3100 Genetic Analyzer. Sequence data were analyzed using Mutation Surveyor V3.25 (Softgenetics, State College, PA, USA). Two sided Fisher's exact test was performed to compare mutation frequencies in primary versus secondary cases, and in the analysis of cooperating mutations.

## Results and Discussion

A total of 84 CN-AML patients were recruited for mutational analysis, including 51 primary cases and 33 cases secondary to either MDS (n = 24) or CMML (n = 9). The 16 genes analyzed were: *ASXL1*, *NPM1*, *FLT3*, *TET2*, *IDH1*, *IDH2*, *RUNX1*, *DNMT3A*, *NRAS*, *JAK2*, *WT1*, *CBL*, *SF3B1*, *TP53*, *KRAS* and *MPL*. The regions analysed for each gene are detailed in Table S2. The frequencies of mutation are shown in Table 1. The most frequently mutated genes in primary cases were *NPM1* (60.8%) and *FLT3* (50.0%), and in secondary cases *ASXL1* (48.5%) and *TET2* (30.3%).

**Table 1:** Frequency of mutations in normal karyotype AML samples.

	All CN-AML samples (n = 84)	Primary AML (n = 51)	Secondary AML (n = 33)		p value
			From MDS (n = 24)	From CMML (n = 9)	
***ASXL1***	**18 (21.4%)**	2 (3.9%)	10 (41.7%)	6 (66.7%)	<0.0001
***NPM1***	**35 (41.7%)**	31 (60.8%)	3 (12.5%)	1 (11.1%)	<0.0001
***FLT3***	**29/81 (35.8%)**	24/48 (50%)	4 (16.7%)	1 (11.1%)	0.0019
***JAK2***	**3/60 (5%)**	0/44	2/15 (13.3%)	1 (11.1%)	0.0404
***TET2***	**21/81 (25.9%)**	11/48 (22.9%)	4 (16.7%)	6 (66.7%)	0.6065
***IDH1***	**10/82 (12.2%)**	7/50 (14.0%)	3/23 (13.0%)	0	0.7327
***IDH2***	**10/82 (12.2%)**	7/50 (14.0%)	2/23 (8.7%)	1 (11.1%)	0.7327
***RUNX1***	**12/81 (14.8%)**	6 (11.8%)	4/21 (19%)	2 (22.2%)	0.3553
***CBL***	**2 (2.4%)**	2 (3.9%)	0	0	0.5172
***MPL***	**0**	0	0	0	1
***TP53***	**1 (1.2%)**	0	1 (4.2%)	0	0.3929
***NRAS***	**5 (6%)**	0	3 (12.5%)	2 (22.2%)	0.0077
***KRAS***	**0**	0	0	0	1
***WT1***	**3 (3.6%)**	3 (5.9%)	0	0	0.2758
***DNMT3A***	**14 (16.7%)**	13 (25.5%)	1 (4.2%)	0	0.0068
***SF3B1***	**2 (2.4%)**	0	2 (8.3%)	0	0.1515
***FLT3*** ** mutations breakdown**					
***FLT3-ITD***	**27/81 (33.3%)**	23/48 (47.9%)	3 (12.5%)	1 (11.1%)	0.0008
***FLT3-TKD*** *	**3/81 (3.7%)**	2/48 (4.2%)	1 (11.1%)	0	1
***IDH2*** ** mutations breakdown**					
***IDH2-R140Q***	**9/82 (11.0%)**	6/50 (12.0%)	2/23 (8.7%)	1 (11.1%)	1
***IDH2-R172K***	**1/82 (1.2%)**	1/50 (12.0%)	0	0	1

* One primary AML sample presented concomitant *FLT3-ITD* and *FLT3-TKD* mutations.

**Figure 1:**
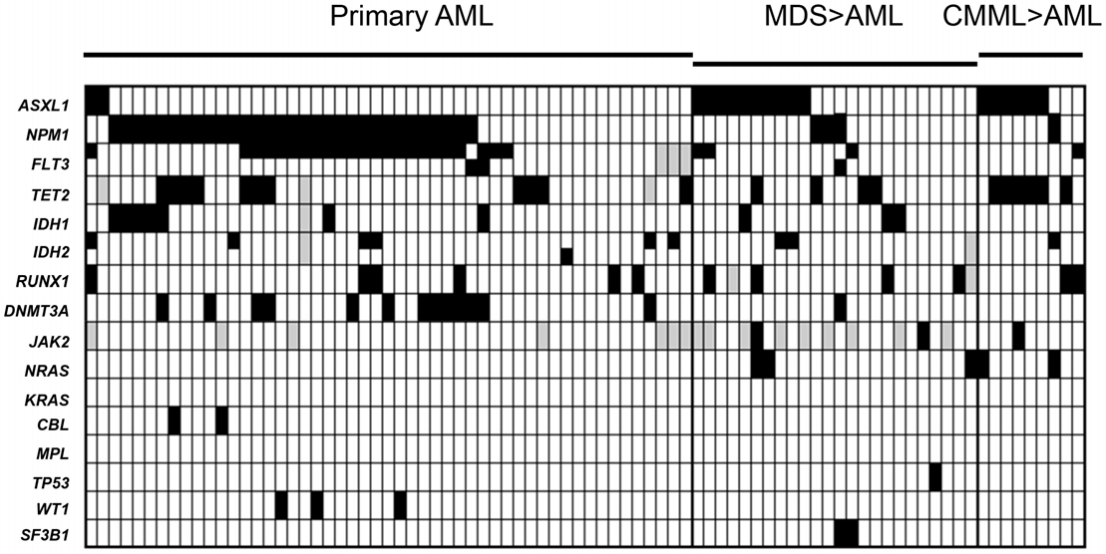
Concurrence of mutations in 16 genes analyzed in CN-AML samples. Columns show results for each of the 84 analysed cases. Solid boxes indicate mutated cases. Grey boxes mark unavailable data. *FLT3-ITD* mutations are indicated with top-half solid boxes and *FLT3-TKD* with bottom-half solid boxes. Similarly, *IDH2-R140Q* mutations are shown with top-half solid boxes and *IDH2-R172K* with bottom-half solid boxes.

An analysis of the mutations occurring in more than 10% of cases revealed statistically significant associations (Figure 1, Table S3). In agreement with previous reports, *FLT3* and *DNMT3A* mutations were significantly associated with *NPM1* mutations, [12] whereas patients with *ASXL1* mutations had significantly lower incidence of *NPM1* and *DNMT3A* mutations. [8], [9] *IDH1* and *IDH2* mutations were mutually exclusive. With the exception of one patient, no cases with IDH1/2 mutation also had a *TET2* mutation. *IDH1* and *IDH2* mutations were less frequent in *TET2*-mutated than in *TET2*-wt patients, and this has been reported before. [19], [20] Concurrence of *IDH1/2* and *ASXL1* mutations was also a relatively infrequent event in our patient cohort (Figure 1). This observation is in agreement with a report on a series of 63 AML secondary to MPN cases. [3]

*ASXL1* mutations were significantly more frequent in secondary AML compared to *de novo* AML cases (primary cases: 2/51, 3.9%; secondary to MDS/CMML: 16/33, 48.5%, p<0.0001). We have previously reported a high prevalence of *ASXL1* mutations in advanced MDS. [18] *NPM1*, *FLT3*, and *DNMT3A* mutations were significantly more common in primary CN-AML than in secondary AML cases (Table 1). *NRAS*, *JAK2*, *SF3B1* and *TP53* mutations were exclusively present in secondary AML samples (Table 1). Only 9.5% of the samples analyzed (8/84, 6 *de novo* and 2 post-MDS cases) showed no mutation in any of the genes tested. When considering only the *ASXL1*, *NPM1*, *FLT3*, *TET2*, *IDH1/2* and *RUNX1* gene analysis, 88% of *de novo* CN-AML included in this series presented at least one molecular marker. For secondary cases, 85% of patients carried mutations in at least one of these 7 genes.

Recent reports showed that *DNMT3A* mutations are associated with a poor outcome in AML, [21], [22] and that the location of the mutations could have an impact in age-related risk classification. [23] It is worth noting that in our series, *DNMT3A* was not found as a sole mutation suggesting that additional aberrations are needed to sustain leukemogenic development.

Approximately 70% of CN-AML cases secondary to either MDS or CMML presented mutations in at least one of *ASXL1*, *TET2*, *IDH1* or *IDH2* genes. Therefore, we chose to assess the presence and chronology of *ASXL1*, *TET2* and *IDH1/2* mutational events, in order to investigate whether they could have a role in disease development or evolution. We studied 15 MDS and 4 CMML cases that progressed to AML, for which at least two samples at different time-points were available. Remarkably, with the exception of two patients all of them possessed at least one gene mutation at some stage of the disease. The majority showed the same mutations at early and later stages of the disease, except one patient who developed an *IDH1* mutation at transformation, a second patient with a *TET2* mutation who acquired an additional *ASXL1* mutation at transformation, and another patient who developed a nonsense mutation of *ASXL1* at AML stage, and showed rapid disease evolution (Table 2). On the basis of this study we therefore did not find any consistent patterns in mutation acquisition. A sensitive mutation analysis (such as allele-specific PCR) at early stages of AML in future studies could help clarify whether the mutations found in cases from later stage AML were already present as a minor pre-existing clone at the earlier stage, and if so, how it evolved as the disease progressed to AML.

**Table 2:** Mutational analysis of serial samples from patients in transformation.

Sample ID	Disease stage	Age	Time from diagnosis (+months)	Cytogenetics	ASXL1	TET2	IDH1	IDH2	Comments
Patient #1	CMML	58	0	46,XY[30]	wt	M1388I	wt	wt	
	AML		(+45)	68,XXYYY,+3,+4,+der(6)x3,+7,+8,+8,+9,+11,+11,+12,+13,+17,+19,+19,+20,+21,+21,+22[9]/46,XY[41]	wt	Q1445X	wt	wt	Previously to this sampling, he was treated with azacitidine. After treatment, the original clone was replaced with an aberrant karyotype new one, now harbouring a different *TET2* mutation.
Patient #2	CMML	65	0	46,XY[30]	2242C>CT; Q748X+2863A>AT; T957S	No mut	wt	wt	
	AML		(+9)	46,XY,i(17)(q10)[15]/46,XY[15]	2242C>CT; Q748X+2863A>AT; T957S	No mut	wt	wt	
Patient #3	CMML	65	0	47,XY,+8,add(21)(q22)[30]	1902–1924delAGAGGCGGCCACCACTGCCATCG; E635RfsX15	2959_2962het_dupAGAC+2954_2957het_dupAAAC	wt	wt	
	AML		(+6)	47,XY,+8,add(21)(q22)[18]/46,XY,+8,−21,add(21)(q22)[7]	wt	wt	wt	wt	Previously to this sampling he underwent QT and BMT. The initial clone remitted, but after 5 months a new clone appeared, now including Monosomy 21, and having lost the previously detected mutations.
Patient #4	CMML	42	0	45,XY,−7[4]/46,XY[21]	wt	wt	wt	wt	
	AML		(+5)	45,XY,−7[47]/46,XY[3]	wt	wt	wt	wt	
Patient #5	MDS	66	0	46,XY[30]	1902–1924delAGAGGCGGCCACCACTGCCATCG; E635RfsX15	wt	wt	wt	
	AML		(+5)	46,XY[30]	1902–1924delAGAGGCGGCCACCACTGCCATCG; E635RfsX15	wt	wt	wt	
	AML		(+10)	46,XY[30]	1902–1924delAGAGGCGGCCACCACTGCCATCG; E635RfsX15	wt	wt	wt	Sample after azacitidine treatment.
	AML		(+12)	46,XY[30]	1902–1924delAGAGGCGGCCACCACTGCCATCG; E635RfsX15	wt	wt	wt	Sample after azacitidine treatment.
Patient #6	MDS	72	0	46,XY[30]	1925het_insA; G643RfsX13	L34F	wt	wt	
	AML		(+11)	46,XY[30]	1925het_insA; G643RfsX13	L34F	wt	wt	Additional *JAK2*, *RUNX1* and *NRAS* mutations.
Patient #7	MDS	70	0	46,XY[30]	1934dupG; G646WfsX12	wt	wt	wt	
	MDS (RAEB)		(+8)	46,XY[30]	1934dupG; G646WfsX12	wt	wt	wt	
	AML		(+15)	46,XY[30]	1934dupG; G646WfsX12	wt	wt	wt	Additional *NRAS* mutation.
Patient #8	MDS	76	0	46,XX[30]	1902–1924delAGAGGCGGCCACCACTGCCATCG E635RfsX15	wt	wt	wt	
	AML		(+12)	46,XX[30]	1902–1924delAGAGGCGGCCACCACTGCCATCG E635RfsX15	wt	R132C	wt	
Patient #9	MDS (RA)	77	0	46,XX[30]	wt	Q403X	wt	wt	
	CMML		(+8)	46,XX[30]	1934dupG G646WfsX12	Q403X	wt	wt	AML-transformed at (+40).
Patient #10	MDS (RA)	ND	0	46,XX[30]	1934dupG G646WfsX12	wt	wt	wt	
	CMML		(+12)	46,XX[30]	1934dupG G646WfsX12	ND	wt	wt	
Patient #11	MDS	84	0	46,XX[30]	1934dupG G646WfsX12	L1151P	wt	wt	
	MDS (RAEB)		(+12)	46,XX[30]	1934dupG G646WfsX12	L1151P	wt	wt	
Patient #12	MDS	76	0	46,XX[30]	1934dupG G646WfsX12	Y867H	wt	R140Q	
	MDS (RAEB)		(+3)	46,XX[30]	1934dupG G646WfsX12	Y867H	wt	R140Q	Previously to this sample, she underwent one course of AraC.
Patient #13	MDS	64	0	46,XX[30]	1934dupG	wt	wt	R140Q	
	MDS (RAEB)		(+12)	46,XX[30]	1934dupG	wt	wt	R140Q	
Patient #14	MDS	79	(+20)	46,XX,del(5)(q15∶q33)[30]	1934dupG G646WfsX12	S794X	wt	wt	
	MDS (RAEB)		(+53)	46,XX,del(5)(q15∶q33)[30]	1934dupG G646WfsX12	S794X	wt	wt	
Patient #15	MDS	80	0	46,XX[30]	wt	wt	wt	wt	
	MDS		(+1)	46,XX[30]	wt	wt	wt	wt	
	AML		(+2)	46,XX[30]	2893C>C/T; R965X	wt	wt	wt	Non mutated for any of the 15 other genes included in this study.
Patient #16	MDS	71	0	45,XX,add(3)(p14),del(5)(q13∶q33),add(7)(q22),+8,−12,−13,−16,i(20)(q10),+mar1[18]/48,XX,+1,del(5)(q13∶q33),add(7)(q22),+8,−12,−13,+mar1,+mar2[7]	wt	wt	wt	wt	
	MDS (RAEB)		(+6)	45,XX,add(3)(p14),del(5)(q13∶q33),add(7)(q22),+8,−12,−13,−16,i(20)(q10),+mar1[18]/48,XX,+1,del(5)(q13∶q33),add(7)(q22),+8,−12,−13,+mar1,+mar2[7]	wt	wt	wt	wt	Later (+10), she developed four additional complex karyotype clones.
Patient #17	MDS (RAEB)	65	0	46,XY[30]	1863–1873del L622RfsX9	wt	wt	wt	
	AML		(+10)	46,XY[30]	1863–1873del L622RfsX9	wt	wt	wt	Additional *FLT3* mutation (ITD)
Patient #18	MDS (RA)	ND	0	45,XX,−7[30]	1934dupG G646WfsX12 (same mutation as M871)	wt	wt	wt	
	AML		(+8)	45,XX,−7[30]	1934dupG G646WfsX12	wt	wt	wt	
Patient #19	MDS	ND	0	46,XX[30]	wt	wt	wt	R140Q	
	MDS (RAEB)		(+9)	46,XX[30]	wt	wt	wt	R140Q	

In order to investigate whether the observed low incidence of *ASXL1* mutations is a specific characteristic of karyotypically normal *de novo* cases, or is a common feature of other subtypes of primary AML, we screened an additional cohort of 100 primary AML, including the most common karyotypic subgroups. Overall, only 8 out of 100 cases showed nonsense or frameshift mutations (8%) (Table S4), confirming that *ASXL1* mutations are less common in primary AML than in secondary AML.

This report concerns the analysis of the largest number of gene mutations in CN-AML studied to date. Our results show that 85% of CN-AML patients have mutations in one or more of 7 selected genes (*ASXL1*, *NPM1*, *FLT3*, *TET2*, *IDH1/2* and *RUNX1*). This finding will facilitate further analysis of this important group of patients by enabling CN-AML patients to be subdivided into groups with common mutation patterns. Detailed studies of the CN-AML subgroups in regard to their hematological features, prognosis, disease progression and treatment response will now be facilitated.

## Supporting Information

Table S1 Relevant literature on mutations of AML patients. ND = not done. CN = cytogenetically normal. MPN = myeloproliferative neoplasm. Yo = years old. CBF = core binding factor. APL = acute promyelocytic leukemia.
(PDF)

Table S2 Primers and PCR conditions. PCR was performed using ThermoStart PCR Master Mix (Thermo Fisher Scientific), following the manufacturer's protocol, 35 cycles, unless otherwise stated, using indicated annealing temperature. The same primers were used for Sanger sequencing unless otherwise stated.
(PDF)

Table S3 Double-sided Fisher's exact test analysis of cooperation between most frequent mutations in normal karyotype AML samples.
(PDF)

Table S4 *ASXL1* mutations in 100 de novo AML cases with aberrant cytogenetics.
(PDF)
