# Supplementary material for: Mutation Patterns of 16 Genes in Primary and Secondary Acute Myeloid Leukemia (AML) with Normal Cytogenetics
Source: PLoS One. 2012 Aug 9;7(8):e42334. doi: 10.1371/journal.pone.0042334 (PMC3415392; doi:10.1371/journal.pone.0042334)
Supplement: Table S1 — Relevant literature on mutations of AML patients. ND = not done. CN = cytogenetically normal. MPN = myeloproliferative neoplasm. Yo = years old. CBF = core binding factor. APL = acute promyelocytic leukemia. (PDF) [file pone.0042334.s001.pdf]

Table S1. Relevant literature on mutations of AML patients. ND = not done. CN = cytogenetically normal. MPN = myeloproliferative neoplasm. Yo = years old. CBF = core binding factor. APL = acute promyelocytic leukemia.

Reference	Samples analysed	***ASXL1***	***NPM1***	***FLT3***	***JAK2***	***TET2***	***IDH1/2***	***AML1/RUNX1***	***CBL***	***MPL***	***TP53***	***NRAS***	***KRAS***	***WT1***	***DNMT3A***	***SF3B1***
Present study	84 NC-AML (51 *de novo* and 33 secondary to MDS/CMML)<br>*De novo* AML with altered karyotype (n=100)	18/84 (21.4%)<br>Primary cases: 10/151 (6.6%)<br>s-AML: 16/33 (48.5%)	35/84 (41.7%)	29/81 (35.8%)	3/60 (5%)	21/81 (25.9%)	21/82 (24.4%)	12/81 (14.8%)	2/84 (2.4%)	0/84	1/84 (1.2%)	5/84 (6%)	0/84	3/84 (3.6%)	14/84 (16.7%)	2/84 (2.4%)
Rocquain *et al.* BMC Cancer. 2010. Reference 2	AML post MDS (n=64) without balanced translocation or complex karyotype	11/64 (17.2%)	28/64 (43.7%)	19/64 (29.7%)	1/64 (1.7%)	9/64 (14.1%)	18/64 (28.1%)	9/64 (14.1%)	1/64 (1.7%)	ND	ND	3/64 (4.7%)<br>(not specified whether mutations are on NRAS or KRAS)	?	3/64 (4.7%)	ND	ND
Abdel-Wahab *et al*. Cancer Res. 2010. Reference 3	AML post MPN (n=63)	19.3%	ND	ND	36.8%	26.3%	9.5%	ND	ND	ND	ND	ND	ND	ND	ND	ND
Ishikawa *et al*. Eur J Haematol. 2009. Reference 4	*De novo* AML (n=144; 54 of them CN)	ND	29 / 144, (20.1%)<br>19 / 54, (35.2%)	35 / 144, (24.3%)<br>15 / 54, (27.8%)	ND	ND	ND	3/144 (2.1%)<br>1/54 (1.9%)	ND	ND	11/144 (7.6%)<br>1/54 (1.9%)	8/144 (5.6%)<br>0/54	ND	ND	ND	ND
Abbas *et al*. Haematologica. 2008. Reference 5	*De novo* AML. All karyotypic groups (n=319)	ND	ND	123/319 (38.6%)	ND	ND	ND	ND	2/319 (0.6%)	ND	ND	26/319 (8.6%)	0/319	ND	ND	ND
Couronne *et al.* Leukemia. 2010. Reference 6	AML secondary to MPN (n=19)	ND	ND	ND	10/19 (52.6%)	6/19 (32%)	ND	ND	ND	ND	ND	ND	ND	ND	ND	ND
Schlenk *et al*. N Engl J Med. 2008. Reference 7	CN-AML (n=872)<br>Adults younger than 60 yo	ND	462/872 (53%)	366/872 (42%)	ND	ND	ND	ND	ND	ND	ND	113/872 (13%)	ND	ND	ND	ND
Carbuccia *et al.* Leukemia. 2010. Reference 8	CN-AML (n=46)<br>AML Trisomy 8 (n=14)<br>Other karyotypes (n=3)<br>- - -<br>Primary (n=46) and secondary to myeloid disease (n=17) cases	3/46 (6%)<br>9/17 (53%)	26/46 (56.5%)<br>2/17 (12%)	17/46 (37%)<br>2/17 (12%)	ND	ND	ND	ND	ND	ND	ND	ND	ND	ND	ND	ND
Chou *et al.* Blood. 2010. Reference 9	*De novo* AML (n=501)	54/501 (10.8%)<br>CN: 8.9%<br>Abnormal karyotype: 12.9%	2/54 (3.7%)<br>(shown for ASXL1 mutated cases only)	9/54 (16.7%)<br>(shown for ASXL1 mutated cases only)	ND	ND	ND	16/54 (29.6%)<br>(shown for ASXL1 mutated cases only)	ND	ND	ND	6/54 (11.1%)<br>(shown for ASXL1 mutated cases only)	ND	ND	ND	ND
Flach *et al.* Leukemia. 2011. Reference 10	AML secondary to MDS (n=38, 27 of them were CN)	ND	5/38 (13.2%)	1/38 (2.6%)	ND	ND	ND	12/38 (31.6%)	ND	ND	ND	4/38 (10.5%)	ND	ND	ND	ND
Dicker *et al*. Leukemia. 2010. Reference 11	AML secondary to MDS (n=101, 54 of them were CN)	ND	8/94 (8.5%)	12/101 (11.9%)	ND	ND	ND	28/101 (27.7%)	ND	ND	ND	8/73 (11.0%)	ND	ND	ND	ND
Shen *et al.* Blood. 2011. Reference 12	AML (n=1185)<br>Subset of 605 cases without prognostic cytogenetic markers except for 11q23	27/605 (5.2%)	122/605 (20.9%)	61/605 (10.8%)<br>APL 13.4%	ND	65/605 (12.7%)	IDH1 52/605 (9.3%)<br>IDH2 53/605 (9.8%)	ND	ND	ND	ND	34/605 (5.9%)<br>CBF AML 44/452 (9.7%)<br>APL 5.4%	ND	20/605 (3.7%)	73/605 (12.3%)	ND

Thol *et al*. J Clin Oncol. 2011. Refence 13	AML (n=489)<br>Younger than 60 yo<br>All karyotypes excluding PML-RARA or t(15;17)<br>- - -<br>De *novo* AML (n=438)<br>secondary AML (n=51)	ND	56/84 (66.7%) (shown for DNMT3A mutated cases only)	34/85 (40%) (shown for DNMT3A mutated cases only)	ND	ND	IDH1 13/79 (16.5%)<br>IDH2 9/79 (11.4%)<br>(shown for DNMT3A mutated cases only)	ND	ND	ND	ND	8/77 (10.4%) (shown for DNMT3A mutated cases only)	ND	15/80 (18.7%) (shown for DNMT3A mutated cases only)	87/489 (17.8%)<br>CN-AML 27.2%	ND
Beer *et al.* Blood. 2010. Reference 14	AML post MPN (n=16)	ND	ND	1/16 (6.2%)	7/16 (43.7%)	2/16 (12.5%)	ND	6/16 (37.5%)	1/16 (6.2%)	ND	4/16 (25%)	2/16 (12.5%)	ND	ND	ND	ND
Pratcorona *et al*. Haematologica. 2011. Reference 15	AML (n=882), including 390 CN-AML	*de novo* AML 36/795 (4.5%)<br>AML post-MDS 3/27 (11.1%)<br>Therapy-related AML 4/40 (10%)<br>CN-AML 21/390 (5.4%)	0/259 (shown for ASXL1 mut cases only)	FLT3-ITD 4/204 (2%)<br>FLT3-TKD 2/75 (2.7%)<br>(shown for ASXL1 mut cases only)	ND	ND	IDH1 4/55 (7.3%)<br>IDH2 5/96 (5.2%)<br>(shown for ASXL1 mut cases only)	ND	ND	ND	ND	7/93 (7.5%) (shown for ASXL1 mut cases only)	ND	ND	ND	ND
Metzeler *et al*. Blood. 2011. Reference 16	Primary CN-AML (n=423)	38/429 (8.9%)	2/38 (5.3%) (shown for ASXL1 mut cases only)	FLT3-ITD 4/38 (10.5%)<br>FLT3-TKD 2/38 (5.3%) (shown for ASXL1 mut cases only)	ND	11/38 (28.9%) (shown for ASXL1 mut cases only)	16/38 (42.1%) (shown for ASXL1 mut cases only)	ND	ND	ND	ND	ND	ND	1/38 (2.6%) (shown for ASXL1 mut cases only)	ND	ND
Jankowska *et al*. Blood. Reference 17	AML secondary to CMML (n=19), including 11 CN-AML	5/19 (26.3%)<br>1/11 (9.1%)	ND	ND	ND	11/19 (57.9%)<br>5/11 (45.5%)	1/19 (5.3%)<br>0/11	ND	3/19 (15.8%)<br>1/11 (9.1%)	ND	ND	2/19 (10.5%)<br>1/11 (9.1%)	1/19 (5.3%)<br>0/11	ND	5/19 (26.3%)<br>4/11 (36.4%)	ND
