# Supplementary material for: Mutation Patterns of 16 Genes in Primary and Secondary Acute Myeloid Leukemia (AML) with Normal Cytogenetics
Source: PLoS One. 2012 Aug 9;7(8):e42334. doi: 10.1371/journal.pone.0042334 (PMC3415392; doi:10.1371/journal.pone.0042334)
Supplement: Table S2 — Primers and PCR conditions. PCR was performed using ThermoStart PCR Master Mix (Thermo Fisher Scientific), following the manufacturer's protocol, 35 cycles, unless otherwise stated, using indicated annealing temperature. The same primers were used for Sanger sequencing unless otherwise stated. (PDF) [file pone.0042334.s002.pdf]

Table S2. Primers and PCR conditions. PCR was performed using ThermoStart PCR Master Mix (Thermo Fisher Scientific), following manufacturer protocol, 35 cycles, if not stated otherwise, using indicated annealing temperature. Same primers were used for Sanger sequencing if non stated otherwise.

amplicon	sequence oligonucleotide Forward 5'-3'	sequence oligonucleotide Reverse 5'-3'	Size PCR product (bp)	PCR conditions	Seq primers	References / comments
ASXL1 exon 12 PCR1	AGGTCAGATCACCCAGTCAGTT	TAGCCCATCTGTGAGTCCAACTGT	561	60		Gelsi-Boyer et al. Br J Haematol. 2009 Jun;145(6):788-800
ASXL1 exon 12 PCR2	AGAGGACCTGCCTTCTCTGAGAAA	TTCGATGGGATGGGTATCCAATGC	558	60		
ASXL1 exon 12 PCR3	ACTTGAAAACCAAGGCTCTCGT	GCAACCATCCCATCTGTCCTTGTA	532	60		
ASXL1 exon 12 PCR4	GGTGGACAAGGATGAGAAACCCAA	TGTCCTGTGACATAGCACGGACTT	674	60		
ASXL1 exon 12 PCR5	TGGATTCCAAAGAGCAGTTCTCTTC	CATGACAAAGGGCATCCCTTCCAA	533	60		
ASXL1 exon 12 PCR6	ACAGGAAAGCTACTGGGCATAGTC	CAAGAGTGCTCCTGCCTAAAGAGT	593	60		
NPM1 Exon 12	TTAACTCTCTGGTGGTAGAATGAA	CAAGACTATTTGCCATTCCTAAC	558	AmpliTaq Gold DNA Pol (PE Biosystems, Foster City, CA), 55	GGCATTTTGGACAACACA	Döhner *et al*. Blood. 2005; 106(12):3740-3746
FLT3 Exon 11	CAATTTAGGTATGAAAGCC	CAAACTCTAAATTTTCTCT	133-366	AmpliTaq Gold DNA Pol (PE Biosystems, Foster City, CA), 55		Nakao *et al.* Leukemia. 1996; 10:1911-1918
FLT3 Exon 17	CCGCCAGGAACGTGCTTG	GCAGCCTCACATTGCCCC	114	AmpliTaq Gold DNA Pol (PE Biosystems, Foster City, CA), 55	EcoR V Digestion	
JAK2 Exon 14	TCCTCAGAACGTTGATGGCAG	ATTGCTTTCCTTTTTCACAAGAT	453	59		Jones *et al*. Blood. 2005; 106(6):2162-2168
JAK2 Exon 14 mutant allele T	TCCTCAGAACGTTGATGGCAG	GTTTTACTTACTCTCGTCTCCACAAAA	279	59	ARMS PCR (see ref)	
JAK2 Exon 14 mutant allele G	ATTGCTTTCCTTTTTCACAAGAT	GCATTTGGTTTTAAATTATGGAGTATATG	229	59		
TET2 exon 3 PCR1	TGAACTTCCCACATTAGCTGGT	GAAACTGTAGCACCATTAGGCATT	955	55	GATAGAAATAAACACATTTT	Gelsi-Boyer *et al*. Br J Haematol. 2009 Jun;145(6):788-800
TET2 exon 3 PCR2	CAAAAGGCTAATGGAGAAAGACGTA	GCAGAAAAGGAATCCTTAGTGAACA	836	55		
TET2 exon 3 PCR3	GCCAGTAAACTAGCTGCAATGCTAA	TGCCTCATTACGTTTTAGATGGG	846	55		
TET2 exon 3 PCR4	GACCAATGTCAGAACACCTCAA	TTGATTTTGAATACTGATTTTCACCA	867	60		
TET2 exon 3 PCR5	TTGCAACATAAGCCTCATAAACAG	ATTGGCCTGTGCATCTGACTAT	788	60		
TET2 exon 3 PCR6	GCAACTTGCTCAGCAAAGGTACT	TGCTGCCAGACTCAAGATTTAAAA	781	60		
TET2 exon 4	ATACTACATATAATACATTCTAATTCCCTCACTG	TGTTTACTGCTTTGTGTGTGAAGG	495	55		
TET2 exon 5	CATTTCTCAGGATGTGGTCATAGAAT	CCCAATTCTCAGGGTCAGATTTA	286	55		
TET2 exon 6	AGACTTATGTATCTTTCATCTAGCTCTGG	ACTCTCTTCCTTTCAACCAAAGATT	599	60		
TET2 exon 7	ATGCCACAGCTTAATACAGAGTTAGAT	TGTCATATTGTTCACTTCATCTAAGCTAAT	362	55		
TET2 exon 8	GATGCTTTATTTAGTAATAAAGGCACCA	TTCAACAATTAAGAGGAAAAGTTAGAATAATATTT	354	55		
TET2 exon 9	TGTCATTCCATTTTGTTTCTGGATA	AAATTACCCAGTCTTGCATATGTCTT	361	55		
TET2 exon 10	CTGGATCAACTAGGCCACCAAC	CCAAAATTAACAATGTTCATTTTACAATAAGAG	774	55		
TET2 exon 11 PCR1	GCTCTTATCTTTGCTTAATGGGTGT	TGTACATTTGGTCTAATGGTACAACTG	748	60		
TET2 exon 11 PCR2	AATGGAAACCTATCAGTGGACAAC	TATATATCTGTTGTAAGGCCCTGTGA	1107	60		
IDH1 Ex 4	TGTGTTGAGATGGACGCCTATTTG	TGCCACCAACGACCAAGTCA	481	55		Thol F *et al.* Haematologica. 2010 Oct;95(10):1668-74.
IDH2 Ex 4	GGGGTTCAAATTCTGGTTGA	CTAGGCGAGGAGCTCCAGT	290	53		
RUNX1 Ex 3	GCTGTTTGCAGGGTCCTAA	CCTGTCCTCCCACCACCCTC	340	55 (5% DMSO)		COSMIC database (Wellcome Trust Sanger Institute, http://www.sanger.ac.uk/genetics/CGP/cosmic/) shows 100% of *RUNX1* somatic mutations in haematopoietic neoplasms within this region (out of 366 reported mutations)
RUNX1 Ex 4	CATTGCTATTCCTCTGCAACC	TGCCATGAAACGTGTTTCAAGC	336	56		
RUNX1 Ex 5	TCAGGCCACCAACCTCATTCTG	CCAGCCCCAAGTGGATGCAC	177	60		
RUNX1 Ex 6	AGCCCCAGTTTTAGGAAATCCAC	GAGCATCAAGGGGAAACCCC	401	58		
RUNX1 Ex 7b	CCCACCCCACTTTACATATAATTG	CCAGCTCAGCTGCAAAGAATGTG	245	57		
RUNX1 Ex 8	CCGCAACCTCCTACTCACTT	GCTTGTCGCGAACAGGAG	604	57 (10% DMSO)		
c-CBL Ex 7	CTTACACCACGTTGCCCTTT	TGGGTCCTATTTTAAGCTCCA	364	57		Sanada *et al*. Nature. 2009 Aug 13;460(7257):904-8
c-CBL Ex 8	AGGACCCAGACTAGATGCTTTC	GGCCACCCCTTGTATCAGTA	386	59		
c-CBL Ex 9	TACTGATACAAGGGGTGGCC	TCGTTAAGTGTTTTACGGCTTT	399	59		
MPL Exs 9-10	ATTCGGAGCTGCAGGATTT	ACAGAGCGAACCAAGAATGC	683	59	Mentioned primers were used for sequencing, plus AGTAGGGGCTGGCTGGATGA (for sequencing exon 10 from intron 9-10)	Modified from Ding *et al*. Blood. 2009 Oct 8;114(15):3325-8
TP53 Exs 3-4	GTGGGAAGCGAAAATTCCAT	GCCAGGCATTGAAGTCTCAT	506	60		COSMIC database (Wellcome Trust Sanger Institute, http://www.sanger.ac.uk/genetics/CGP/cosmic/) shows 100% of *TP53* somatic mutations in haematopoietic neoplasms within this region (out of 200 reported mutations)
TP53 Exs 5-6	TGTTCACTTGTGCCCTGACT	TTAACCCCTCCTCCCAGAGA	467	56		
TP53 Ex 7	GAGCTTGCAGTGAGCTGAGA	GGGATGTGATGAGAGGTGGA	390	61.5		
TP53 Exs 8-9	GACAAGGGTGGTTGGGAGTA	GCCCCAATTGCAGGTAAAAC	500	56		
NRAS Ex 2	AGAACCAAATGGAAGGTCACA	TGGGTAAAGATGATCCGACA	302	57		COSMIC database (Wellcome Trust Sanger Institute, http://www.sanger.ac.uk/genetics/CGP/cosmic/) shows 100% of *NRAS* somatic mutations in haematopoietic neoplasms within this region (out of 634 reported mutations)
NRAS Ex 3	GCAATTTGAGGGACAAACCA	CCCTAGATTCTCAATGTCAAACAA	325	55		
KRAS Ex 2	CTTAAGCGTCGATGGAGGAG	AGAATGGTCCTGCACCAGTAA	391	57		COSMIC database (Wellcome Trust Sanger Institute, http://www.sanger.ac.uk/genetics/CGP/cosmic/) shows 96.4% of *KRAS* somatic mutations in haematopoietic neoplasms within this region (out of 139 reported mutations)
KRAS Ex 3	TTTTTGAAGTAAAAGGTGCACTG	TGCATGGCATTAGCAAAGAC	316	57		
WT1 Ex 4	TGCAGAGATCAGTGGGATGA	CTGTGGAAAGGCAATGGAAT	284	55		COSMIC database (Wellcome Trust Sanger Institute, http://www.sanger.ac.uk/genetics/CGP/cosmic/) shows 97.4% of *WT1* somatic mutations in haematopoietic neoplasms within this region (out of 196 reported mutations)
WT1 Ex 5	GGCTTTTCACTGGATTCTGG	CCACCAAATGCTACCCTGAT	250	57		
WT1 Ex 6	GAGGGAGGCAAAAACCAACT	AGGAACTAAAGGGCCGGTAA	302	57		
WT1 Ex 7	CAGTGCTCACTCTCCCTCAA	GTGTGAGAGCCTGGAAAAGG	299	59		
WT1 Ex 8	CCTAACAAGCTCCAGCGAAG	TGTGGGGTGTTTCCTTTTCT	410	57		
WT1 Ex 9	GGGGACTGGGGAAATCTAAG	GCCACGCACTATTCCTTCTC	385	59		
DNMT3A Exs 7-8	ATGGTCCCCTTGAGTGTCAG	CATCACCCCAATTCCAGACT	836	56		COSMIC database (Wellcome Trust Sanger Institute, http://www.sanger.ac.uk/genetics/CGP/cosmic/) shows 98.4% of *DNMT3A* somatic mutations in haematopoietic neoplasms within this region (out of 379 reported mutations)
DNMT3A Exs 9-10	CTGTATCTGGTCCCCTCCAG	CTCCCTAAGCATGGCTTTCC	747	56		
DNMT3A Exs 11-12	GGGAACAAGTTGGAGACCAG	GGTCCCATGTCATTCAAACC	490	56		
DNMT3A Ex 13	GTCACAGTGCCTCCCTTTTC	TGGACACAGTCAGCCAGAAG	308	56		
DNMT3A Ex 14	CAGGGCTTAGGCTCTGTGAG	AGGTGTGCTACCTGGAATGG	359	56		
DNMT3A Exs 15-16	CGGTCTTTCCATTCCAGGTA	CATCATTTCGTTTTGCCAGA	614	56		
DNMT3A Ex 17	GACTTGGGCCTACAGCTGAC	CAAAATGAAAGGAGGCAAGG	345	58		
DNMT3A Ex 18-19	CTTCCTGTCTGCCTCTGTCC	ATGAAGCAGCAGTCCAAGGT	552	56		
DNMT3A Exs 19b-20	GCAGCACTGTGCAATATGGT	CTTCCCCACTATGGGTCATC	549	56		
DNMT3A Ex 21	GCGGGGAGTTTGAAGAGAGT	CCACACTAGCTGGAGAAGCA	342	56		
DNMT3A Ex 22	TTTGGTAGACGCATGACCAG	CAGGACGTTTGTGGAAAACA	301	56		
DNMT3A Ex 23	TCCTGCTGTGTGGTTAGACG	CCTCTCTCCCACCTTTCCTC	654	56		
SF3B1 Ex 12	TGGAAATGAACTCATGCTGTCT	TGCAAAGGAAAAGGTCTAGGAG	427	59		Papaemmanuil E *et al.* NEJM. 2011 Oct 13;365(15):1384-95
SF3B1 Exs 13-14	TTCTGTACATGAGCATTTCATCA	GACAGGCTGTGTGTGTACCTCT	701	59		
SF3B1 Ex 15-16	CTGCAGTTTGGCYGAATAGTTG	AAAATTCTGTTAGAACCATGAAACA	551	59		
