# Supplementary material for: Mutation Patterns of 16 Genes in Primary and Secondary Acute Myeloid Leukemia (AML) with Normal Cytogenetics
Source: PLoS One. 2012 Aug 9;7(8):e42334. doi: 10.1371/journal.pone.0042334 (PMC3415392; doi:10.1371/journal.pone.0042334)
Supplement: Table S3 — Double-sided Fisher's exact test analysis of cooperation between most frequent mutations in normal karyotype AML samples. (PDF) [file pone.0042334.s003.pdf]

Table S3. Double-sided Fisher´s exact test analysis of cooperation between most frequent mutations in normal karyotype AML samples.

Gene X	*ASXL1* mutants among gene X mutants vs *ASXL1* mutants among gene X wt (p value)	*NPM1* mutants among gene X mutants vs *NPM1* mutants among gene X wt (p value)	*FLT3* mutants among gene X mutants vs *FLT3* mutants among gene X wt (p value)	*DNMT3A* mutants among gene X mutants vs *DNMT3A* mutants among gene X wt (p value)	*TET2* mutants among gene X mutants vs *TET2* mutants among gene X wt (p value)	*IDH1/2* mutants among gene X mutants vs *IDH1/2* mutants among gene X wt (p value)
NPM1	0/35 vs 18/49 (p<0.0001)	-	-	-	-	-
FLT3	3/29 vs 15/52 (p=0.0924)	21/29 vs 14/52 (p=0.0001)	-	-	-	-
DNMT3A	0/14 vs 18/70 (p=0.0336)	12/14 vs 23/70 (p=0.0005)	11/14 vs 18/67 (p=0.0005)	-	-	-
TET2	6/21 vs 11/60 (p=0.3580)	8/21 vs 26/60 (p=0.7990)	3/20 vs 25/58 (p=0.0307)	3/21 vs 10/60 (p=1.0000)	-	-
IDH1/2	4/20 vs 14/62 (p=1.0000)	10/20 vs 24/62 (p=0.4382)	5/19 vs 23/60 (p=0.4168)	3/20 vs 11/62 (p=1.0000)	1/19 vs 19/61 (p=0.0314)	-
RUNX1	3/12 vs 14/70 (p=0.7061)	3/12 vs 32/70 (p=0.2200)	6/12 vs 23/67 (p=0.3406)	1/12 vs 13/70 (p=0.6806)	2/12 vs 19/67 (p=0.4994)	4/12 vs 16/69 (p=0.4782)
