# Supplementary material for: Mutation Patterns of 16 Genes in Primary and Secondary Acute Myeloid Leukemia (AML) with Normal Cytogenetics
Source: PLoS One. 2012 Aug 9;7(8):e42334. doi: 10.1371/journal.pone.0042334 (PMC3415392; doi:10.1371/journal.pone.0042334)
Supplement: Table S4 — ASXL1 mutations in 100 de novo AML cases with aberrant cytogenetics. (PDF) [file pone.0042334.s004.pdf]

Table S4. *ASXL1* mutations in 100 *de novo* AML cases with aberrant cytogenetics.

	Samples analysed	ASXL1 mutated
Complex karyotype	35	5
del5q/Monosomy 5	10	-
M3 t(15;17)	15	-
M2 t(8;21)	15	-
Inv(16)	12	-
Trisomy 8	5	-
del20q	4	2
del7q/Monosomy 7	4	1
